# Visual findings in children exposed to Zika *in utero* in Nicaragua

**DOI:** 10.1371/journal.pntd.0011275

**Published:** 2023-05-19

**Authors:** Evelin Martinez, Ryan Max, Filemón Bucardo, Elizabeth M. Stringer, Sylvia Becker-Dreps, Christian Toval-Ruíz, Meylin Chavarria, María J. Meléndez-Balmaceda, Carlos Nuñez, Matthew H. Collins, Michael Boivin, Shiara Ortiz-Pujols, Omar Zepeda, Kaitlyn Cross, Emily W. Gower, Natalie M. Bowman, Sara F. Grace

**Affiliations:** 1 Department of Microbiology, Faculty of Medical Science, National Autonomous University of Nicaragua, León (UNAN-León, Managua), Nicaragua; 2 Department of Epidemiology, University of North Carolina at Chapel Hill, Chapel Hill, North Carolina, United States of America; 3 Department of Obstetrics and Gynecology, University of North Carolina at Chapel Hill, Chapel Hill, North Carolina, United States of America; 4 Department of Family Medicine, University of North Carolina at Chapel Hill, Chapel Hill, North Carolina, United States of America; 5 Ophthalmology Clinic, Victoria Mota Hospital, Jinotega, Nicaragua; 6 Department of Medicine, Emory University, Atlanta, Georgia, United States of America; 7 Department of Psychiatry, Michigan State University, East Lansing, Michigan, United States of America; 8 Obesity Medicine Medical Director at Med Express/Optum, Minneapolis, Minnesota, United States of America; 9 Department of Biostatistics, University of North Carolina at Chapel Hill, Chapel Hill, North Carolina, United States of America; 10 Department of Medicine, University of North Carolina at Chapel Hill, Chapel Hill, North Carolina, United States of America; 11 North Carolina Eye Ear Nose and Throat/Duke health Durham, North Carolina, United States of America; Beijing Children’s Hospital Capital Medical University, CHINA

## Abstract

Knowledge regarding the frequency of ocular abnormalities and abnormal visual function in children exposed to Zika virus (ZIKV) *in utero* but born without congenital Zika syndrome (CZS) is limited. We hypothesized that children exposed to ZIKV *in utero* born without CZS may have visual impairments in early childhood. We performed ophthalmic examination between 16 and 21 months of age and neurodevelopment assessment at 24 months of age with the Mullen Scales of Early Learning test (MSEL) on children enrolled in a cohort born to women pregnant during and shortly after the ZIKV epidemic in Nicaragua (2016–2017). ZIKV exposure status was defined based on maternal and infant serological testing. Visual impairment was defined as abnormal if the child had an abnormal ophthalmic exam and/or low visual reception score in the MSEL assessment. Of 124 children included in the analysis, 24 (19.4%) were classified as ZIKV-exposed and 100 (80.6%) unexposed according to maternal or cord blood serology. Ophthalmic examination showed that visual acuity did not differ significantly between groups, thus, 17.4% of ZIKV-exposed and 5.2% of unexposed had abnormal visual function (*p* = 0.07) and 12.5% of the ZIKV-exposed and 2% of the unexposed had abnormal contrast testing (*p* = 0.05). Low MSEL visual reception score was 3.2-fold higher in ZIKV-exposed than unexposed children, but not statistically significant (OR 3.2, CI: 0.8–14.0; *p* = 0.10). Visual impairment (a composite measure of visual function or low MESL visual reception score) was present in more ZIKV-exposed than in unexposed children (OR 3.7, CI: 1.2, 11.0; *p* = 0.02). However, the limited sample size warrants future investigations to fully assess the impact of *in utero* ZIKV exposure on ocular structures and visual function in early childhood, even in apparently healthy children.

## Author summary

Vertical transmission of Zika virus (ZIKV) during pregnancy has the potential to cause vision abnormalities even in the absence of overt ocular structural abnormalities or congenital Zika syndrome. We were able to classify infants’ ZIKV exposure status by applying a serological algorithm of Zika antibodies in maternal and umbilical cord blood samples. We evaluated the ophthalmological abnormalities in children born in Nicaragua during the American ZIKV epidemic of 2015–2017 with no clinical abnormality detected at birth. We performed eye examinations on the children at 16–21 months of age, and neurocognitive testing by the Mullens Scales of Early Learning (MSEL) was performed at 24 months of age. Visual impairment, a composite measure of abnormal visual function and/or visual reception score was significantly more common in ZIKV-exposed than in unexposed children. We found a nonsignificant trend for a higher proportion of children exposed to ZIKV in utero had abnormal visual function and abnormal contrast testing than unexposed children. The visual reception score from the MSEL was also higher in Zika-exposed children, but these relationships did not reach statistical significance. These impairments could have implications for later academic performance, specifically reading. Early intervention may limit the morbidity associated with congenital ZIKV exposure.

## Introduction

Zika virus (ZIKV), first isolated from a monkey in the Zika forest of Uganda in 1947 [1], historically caused sporadic and geographically-limited epidemics in East Africa and Asia. ZIKV came to international attention after being introduced in the Americas in 2015, resulting in over a million documented cases distributed over nearly all American countries [2]. ZIKV is a single stranded RNA virus that belongs to the *Flaviviridae* family with 2 different phylogenetic lineages (African and Asian), of which the Asian lineage gave rise to the sub-lineage that caused the American epidemic [3].

An epidemiological association between maternal ZIKV infection and congenital malformations, including microcephaly and neurological syndromes was established for the first time in the context of the American epidemic [4]. It was subsequently established that ZIKV has a tropism for the developing central nervous system, and infections during pregnancy can cause congenital Zika syndrome (CZS), characterized by microcephaly and other neurological defects, which can cause severe disability or perinatal death [5]. Since then, it has been increasingly appreciated that fetal infection with ZIKV might alter brain development and lead to a spectrum of neurologic abnormalities including cognitive, motor, and sensory deficits present at birth or in early infancy [6].

Ocular structural abnormalities related to congenital ZIKV infection were first described in Brazil in 2016 [7–9]. Affected children may present with a range of ocular sequelae including chorioretinal atrophy, retinal vascular abnormalities and hemorrhages, lens subluxation, bilateral iris colobomas, optic neuritis and optic atrophy, congenital cataracts and glaucoma [10–12]. While structural eye findings are most common in children with CZS, particularly those with microcephaly, it has been noted that a significant percentage of children may have visual impairment but no obvious manifestations of CZS [13–15]. Eye anomalies may even present as the primary or sole manifestation of in utero ZIKV infection [16].

Maternal ZIKV infection in the absence of fetal infection during gestation does not appear to cause abnormal visual function [17], but vertical transmission has the potential to cause vision abnormalities even in the absence of overt ocular structural abnormalities or CZS [16,18,19]. Signs of abnormal visual function may be found using various visual measures including visual acuity, contrast differentiation, peripheral vision testing, shift of gaze, accommodative reflex to near stimuli and also detection of delays or failure to meet visual developmental milestones [20]. In the absence of ocular structural abnormalities, abnormal visual function is presumed to occur through impairment of the visual cortex or abnormal cortical processing of visual information [21].

To assess the real impact of Zika exposure on children visual health, reliable and robust screening methods are still needed, in particular for children born from mothers experiencing subclinical infections.

We hypothesized that children born to mothers infected with ZIKV during pregnancy, without neurological evidence of CZS, might still have visual abnormalities in early childhood. We recruited a cohort of children whose mothers were pregnant during the ZIKV epidemic of 2016 in Nicaragua and followed them for 48 months to assess neurodevelopment. The great majority of children did not show evidence of CZS at birth. The objective of this sub-study was to evaluate the visual function of children born to Nicaraguan mothers during the ZIKV epidemic peak through comprehensive ophthalmological examination and evaluation of visual reception measured by the MSEL at 24 months.

## Materials and methods

### Ethics statement

The study was approved by the Ethics Committee of the National Autonomous University of Nicaragua-León (UNAN-León) (Acta 93, 2016) and the Institutional Review Board of the University of North Carolina at Chapel Hill (Protocol number 16–1402). All mothers signed informed consent during pregnancy to participated in the serological study [22] and parental permission was signed at birth to perform prospective neurological assessment [23] and ophthalmological examination. This study was carried out in accordance with the ethical standards and following good clinical practices.

### Subject

**Fig 1:**
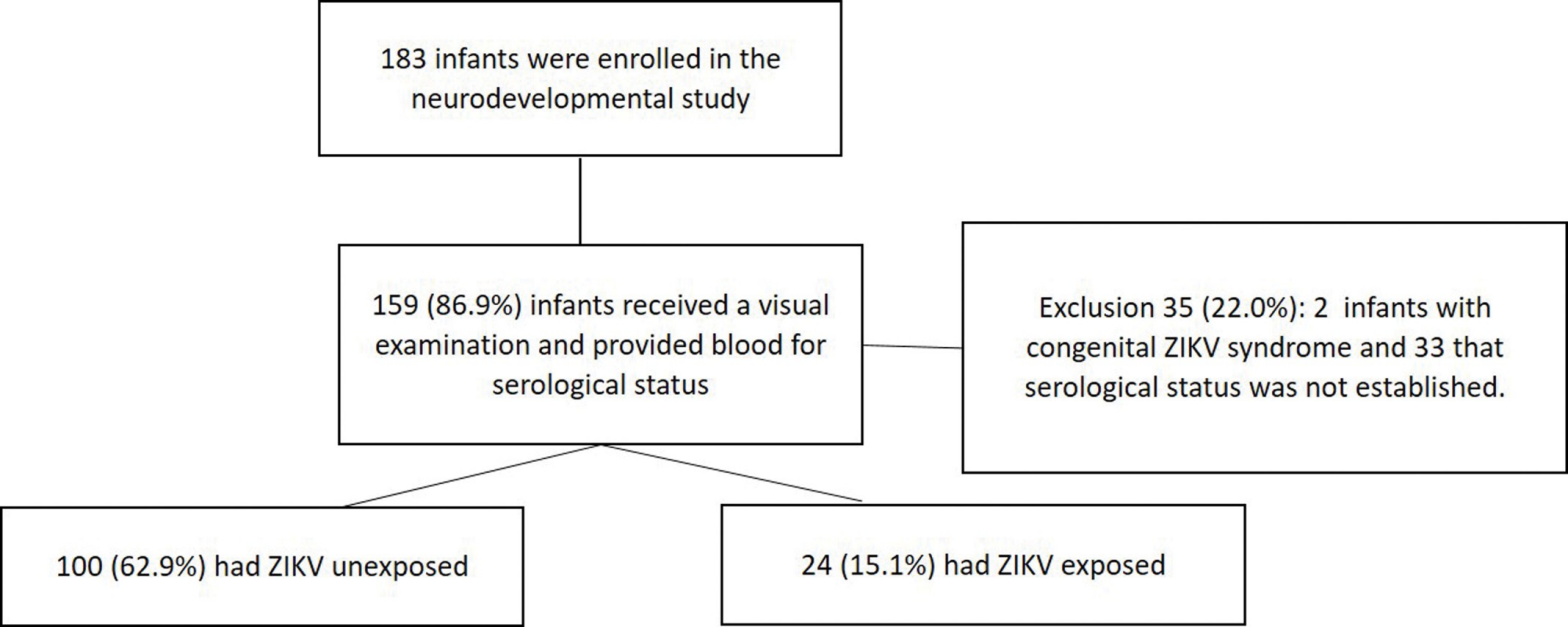


A subset of 124 of 183 children born to mothers who were pregnant during the ZIKV epidemic in León, Nicaragua in 2016 and had no evidence of CZS were followed for 24 months [22,23] (Fig 1). At the time of birth, information was collected regarding type of delivery, Apgar scores, anthropometric measurements, complications during delivery, infant physical examination, and neurological assessment (hypotonia, hypertonia/spasticity, eyes capable of tracking, hyperreflexia, irritability, seizures/tremors and microcephaly) [See S1 Text)].

After discharge from the hospital, children were followed through home visits to assess their neurodevelopment. Medical history and anthropometry of all children were collected at 24 months, and examinations of visual function were performed between 13 and 28 months of age. A poverty index was assigned to each child’s as described elsewhere [24].

### ZIKV serological status

The ZIKV exposure status of the children was established according to mothers’ serological status, as described previously [22]. In brief, children were defined as "ZIKV-exposed” if they met any of the following criteria: 1) high maternal serum titers for ZIKV-neutralizing antibodies (focus reduction neutralization test [FRNT] >3000) at the time of childbirth. 2) a 4-fold or more increase in ZIKV-specific maternal serum FRNT50 value between the prenatal and delivery samples; 3) detection of anti-ZIKV IgM in any maternal or umbilical cord blood serum sample. Children were defined as “ZIKV-unexposed” if the mother had stable ZIKV neutralizing antibody level over the course of the pregnancy (indicative of infection prior to pregnancy) or tested negative for ZIKV neutralizing antibodies (S1 Table).

### Cytomegalovirus and Toxoplasma IgM detection

Umbilical cord serum samples were tested for IgM to cytomegalovirus (Human Gesellschaft fur Biochemica und Diagnotica mbH, Weisbaden, Germany 65205) and *Toxoplasma gondii* (IDG/Sanzay Corporation, Miami, Florida 33169) following the manufacturer’s instructions. A serum was considered IgM-positive using the manufacturer’s cut off value.

### Ophthalmic examination

After informed parental consent, an ophthalmologist performed an initial interview to inquire about the child’s medical history, any parental concerns about the child’s eyes or vision, and any family history of eye disease. Children underwent a complete visual examination by an ophthalmologist collaborating with this study, which consisted of visual acuity testing by preferential looking (LEA GRATINGS, Lea Test Intl LLC, Etters, PA), pupillary examination, ocular motility and alignment, anterior segment examination, cycloplegic refraction and dilated fundus exam. Visual function was evaluated, by shift of gaze between two toys, confrontational visual fields, accommodation, and Hiding Heidi Contrast Test (Good-Lite, Elgin, IL, USA). Confrontational visual fields were considered abnormal if a child did not identify a toy stimulus in all four quadrants (superior, inferior, temporal, and nasal). Accommodation was considered abnormal if the child did not exhibit the accommodation reflex on dynamic retinoscopy. The Hiding Heidi Contrast Test requires children to visually recognize a “Hiding Heidi” Contrast Card when presented next to a blank white card, and the test increases in visual difficulty as Heidi’s contrast gradually decreases from 100% to 1.25%. The examiner looks for a shift of gaze or head turn towards the card with “Heidi”. An abnormal result occurs when the child no longer shifts their gaze in response to a Hiding Heidi contrast card at ≥5% contrast. The examining ophthalmologists were masked to the child’s ZIKV exposure status.

### Mullen Scale for Early Learning

The Mullen Scales of Early Learning (MSEL) [23] is an evaluation of neurological development based on five subscales of cognitive and motor development for ages 0 to 68 months. The five subscale domains correspond to expressive and receptive language, gross and fine motor, and visual reception. The MSEL has excellent correspondence validity with the Bayley Scales of Infant Development, the gold standard to assess neurologic development [24] but is considered more easily adaptable to cross-cultural environments and resource-limited settings [25]. The MSEL’s visual reception scale measures visual perception capacity through the implementation of intra-sensory tasks, which measure discrimination and visual memory through the presentation of shapes and patterns involving oculomotor and visual-motor elements.

The neurodevelopment assessment for this study was performed at 24 months of children’s age. The entire tool consisted of 124 activities performed over 30 to 60 minutes. A raw score was generated, from which a tabulated (T) score was obtained [25]. The sum of the T scores from the 5 domains generated an Early Learning Composite score (ELC) which provided a general measure of the child’s cognitive development. The ELC score from each child fell within one of the following categories: very high (>251), above average (231–251), average (169–230), below average (138–168), very low (80–137). (S2 Table). Study staff administered MSEL evaluations in participants’ homes. The examiners were masked to the ZIKV status of the child. The team consisted of two psychologists and one nurse, who were trained by videoconferences on a weekly basis for 3 months, including virtual sessions and in-person training or in-person workshop. Face-to-face training was carried out periodically throughout the year and videos of the testers administering the MSEL were evaluated by the senior neuropsychologist collaborating on this study.

### Composite outcomes

Two composite outcomes were defined based on the ophthalmologic examination and the neurodevelopment assessment, respectively. These were “abnormal visual function” and “visual impairment”. Visual function was considered abnormal if there were any abnormal findings on the functional eye exam, including abnormal accommodation, abnormal confrontational field testing, absent shift of gaze, or if the Hiding Heidi 5% contrast at 30 cm did not elicit a response. Visual impairment was considered present if children had either abnormal visual function and/or low visual reception score on the MSEL exam (a visual reception subdomain score of ≤ 39). The composite measure of visual impairment was defined to capture all children with potentially abnormal vision due to ocular, neurological, or developmental causes.

### Statistical analyses

Maternal and infant demographic characteristics, as well as birth, neurodevelopmental, and ophthalmologic outcomes were compared between ZIKV-exposed and unexposed infants using chi-squared or Fisher’s exact test for categorical variables and t-tests for continuous variables. Fisher’s exact test was used for comparisons of categorical variables with expected cell counts of ≤ 5, and corresponding exact confidence intervals for odds ratios were calculated using the minimum likelihood principle. While α = 0.05 was considered the cutoff for statistical significance, this study was found to be under-powered to detect modest but clinically relevant effects using the Fisher’s exact test. For example, at the available sample size empirical power is 21% to detect an odds ratio ≠ 1 if the proportion of Zika-exposed infants with a low visual reception (VR) score were double the proportion in unexposed infants (OR ≈ 2.4), and assuming between 8–12% of unexposed infants experience low VR scores. Infants whose ZIKV exposure status was unknown are described in this manuscript, but they were not included in statistical comparisons since we could not definitively categorize their ZIKV exposure. Study data were managed using REDCap and analyzed using SAS software (version 9.0; SAS Institute, Cary, NC), with the exception of exact confidence intervals for odds ratios, which were calculated using the exact2x2 package in R version 4.1 software (R Core team, 2021).

## Results

In this study 124 of 159 enrolled children met the following eligibility criteria: available serological ZIKV status, no CZS at born and completed both the ophthalmologic examination and MSEL evaluation. Of the 124 children included in the analyses, 24 (19.4%) were ZIKV- exposed and 100 (80.6%) ZIKV- unexposed (Fig 1). Sixty-five children (52.4%) were male. Mean birth weight was 3065 grams (SD: ± 492), and 12 (9.7%) were born before 37 weeks’ gestational age. We found no significant difference between ZIKV—exposed and unexposed groups with respect to type of delivery, gestational age, sex, weight at birth and poverty index (Table 1). To rule out any potential bias associated with other congenital infections, cord-blood samples were examined for CMV- and Toxoplasma-IgM. All samples were CMV IgM-negative (0/124), and only 2 (1.7%) of 119 serum available were *Toxoplasma* IgM-positive.

**Table 1:** Description of the characteristics at birth of children exposed and unexposed to ZIKV in a cohort in León, Nicaragua, 2016–2017.

	All participants (%) N = 124	ZIKV Exposed (%) N = 24	ZIKV Unexposed (%) N = 100	Odds Ratio (95% CI)	Mean Difference (95% CI)	p-value
Type of delivery						
Vaginal	69 (55.6)	14 (58.3)	55 (55.0)	1.1 (0.5–2.8)	-------	0.82
Cesarea	55 (44.4)	10 (41.7)	45 (45.0)			
Gestational age						
Preterm	12 (9.7)	2 (8.3)	10 (10.0)	1.2 (0.25–5.9)	-------	>0.99
Term	112 (90.3)	22 (91.7)	90 (90.0)			
Male	65 (52.4)	14 (58.3)	51 (51.0)	1.3 (0.5–3.3)	-------	0.52
Infant birth weight, g, mean (SD)	3065 (492)	3157 (480)	3044 (496)	-------	113 (-109–335)	0.32
**Characteristics of children at the time of examination**						
Age in month, median (IQR)	17.8 (20–16)	19.6 (20–18)	17 (19–15)	-------	1.8 (0.4–3.2)	0.01
WAZ, mean (SD) ^a^	-0.7 (1.5)	-0.9 (1.1)	-0.6 (1.6)	-------	-0.3 (-1.0–0.4)	0.40
HAZ, mean (SD) ^a^	-0.1 (1.2)	-0.1 (1.0)	-0.1 (1.3)	-------	0.0 (-0.6–0.6)	0.91
**Poverty Index** ^b^						
0–1	23 (18.6)	6 (25.0)	17 (17.0)	-------	-------	Ref.
2–3	64 (51.6)	10 (41.7)	54 (54.0)	1.9 (0.6–6.0) 1.3 (0.4–4.3)	-------	0.26
≥ 4	37 (29.8)	8 (33.3)	29 (29.0)			0.88

^a^ WAZ (Wight-for-age Z score) and HAZ (Height-for-age Z score) at 24 month

^b^ Poverty index category: 0–1 Not poor, 2–3 Poor, ≥ 4 Extremely poor

### Ophthalmic examination data

The median age of the participants at the time of the ophthalmic examination was 17.8 months (IQR 20–16). Lea visual acuity did not differ significantly between the two groups (mean difference 0.3, 95% CI -1.0–1.6; *p* = 0.65). We found no significant differences between the exposed and unexposed groups for accommodation, confrontational visual fields or shift of gaze. The prevalence of an abnormal Hiding Heidi contrast test was 12.5% (3/24) in the ZIKV-exposed group and 2.0% (2/100) in the ZIKV-unexposed group (*p* = 0.05). Specific ocular findings among ZIKV- unexposed children were: 1 child (0.8%) had strabismus, 6 (4.8%) had abnormal external eyes, and 2 (1.6%) had anterior segment abnormalities. There were no motility abnormalities nor structural abnormalities in the ZIKV-exposed group. None of the children in our study had structural ocular lesions (Table 2).

Two children were excluded from the analyses because they were born with CZS. The first had severe cortical visual impairment with no reaction to light and no fixation reflex to faces or objects. The second reacted to bright light stimuli, but had inattentive fixation to faces and visual stimuli, an absent accommodation reflex, inability to recognize a toy in any of the four visual quadrants, and inability to visualize the Hiding Heidi contrast test at <25%.

**Table 2:** Ocular finding of children exposed and unexposed to ZIKV in a cohort in León, Nicaragua, 2016–2017.

	All Participants (%) N = 124	ZIKV Exposed (%) N = 24	ZIKV Unexposed (%) N = 100	Odds Ratio (95% CI)	Mean Difference (95% CI)	p-value
**Functional eye exam**						
No change of gaze between 2 objects at 30 cm	4 (3.2)	1 (4.2)	3 (3.0)	1.3 (0.0–12.5)	-------	>0.99
Hiding Heidi Contrast greater than 5%^a^	5 (4.0)	3 (12.5)	2 (2.0)	6.9 (1.0–57.0)	-------	0.05
**Visual acuity**						
Abnormal visual field	0 (0)	0 (0)	0 (0)	-------	-------	-------
Abnormal accommodative reflex	0 (0)	0 (0)	0 (0)	-------	-------	-------
**Lea visual acuity, cpcm (cycles per centimeter of surface), mean (SD)**						
OD^b^	4.6 (1.9)	4.5 (1.9)	4.6 (1.9)	-------	-0.1 (-1.2–1.0)	0.86
OS^c^	4.7 (2.0)	4.5 (1.9)	4.7 (2.0)	-------	-0.2 (-1.3–0.9)	0.71
OU^d^	6.0 (2.8)	6.3 (3.3)	6.0 (2.8)	-------	0.3 (-1.0–1.6)	0.65
No fix and follow	2 (1.6)	0 (0)	2 (2.0)	0.0 (0.0–15.1)	-------	>0.99
No reaction to light	2 (1.6)	0 (0)	2 (2.0)	0.0 (0.0–14.5)	-------	>0.99
**External eye**						
Abnormal motility	0 (0)	0 (0)	0 (0)	-------	-------	-------
Strabismus	1 (0.8)	0 (0)	1 (1.0)	0.0 (0.0–79.2)	-------	>0.99
Abnormal external eye	6 (4.8)	0 (0)	6 (6.0)	0.0 (0.0–3.1)	-------	0.60
Abnormal anterior segment	2 (1.6)	0 (0)	2 (2.0)	0.0 (0.0–16.8)	-------	>0.99
**Evaluation summary**						
Abnormal functional sight	2 (1.6)	1 (4.2)	1 (1.0)	4.3 (0.1–167.3)	-------	0.35
Abnormal refraction	1 (0.8)	0 (0)	1 (1.0)	0.0 (0.0–78.4)	-------	>0.99
Abnormal ocular findings	3 (2.4)	0 (0)	2 (2.0)	0.0 (0.0–14.6)	-------	>0.99

^a^ Values of ≥ 5% of Hiding Heidi contrast card was consider a visual impairment

^b^ Oculus dexter (right eye)

^c^ Oculus sinister (left eye)

^d^ Oculus uterque (both eyes)

### Ophthalmic exam data analyzed with Mullen evaluations

We found no significant differences in the frequency of low ELC scores between the ZIKV-exposed (5/23, 21.7%) and unexposed (18/97, 18.6%) infants (OR 1.2, 95% CI: 0.4, 3.8; *p* = 0.77). The proportion of exposed children with a low visual reception score was higher than of unexposed infants, but the difference did not reach statistical significance (OR 3.2, 95% CI: 0.8, 14.0; *p* = 0.10).

### Composite outcomes

Abnormal visual function (any abnormality on ophthalmological examination and testing) was present in 9/120 children (7.5%). Although the ZIKV-exposed group had a higher proportion of children with abnormal visual function (4/23, 17.4%) than the ZIKV-unexposed (5/97, 5.2%) group, the difference did not reach statistical significance (OR 3.8, 95% CI: 0.9, 15.2; *p* = 0.07) (Table 3).

Visual impairment (a composite score defined as either a low visual reception MSEL score or any adverse finding on the functional eye exam), was present in the 17 (14.2%) children. Visual impairment was present in a larger number of ZIKV-exposed children (7/23, 30.4%) than unexposed children (10/97, 10.3%) (OR 3.7, 95% CI: 1.2, 11.0; *p* = 0.02) (Table 3).

Sensitivity analysis including the group of children with unknown serological status did not influence the trends reported in this study nor the ***association*** between visual impairment and in utero ZIKV exposure (S3 and S4 Tables).

**Table 3:** Scores of Mullen Scale of Early Learning and visual impairment of children exposed and unexposed to ZIKV in a cohort in León, Nicaragua, 2016–2017.

	All participants (%) N = 120	ZIKV Exposed (%) N = 23	ZIKV Unexposed (%) N = 97	Odds Ratio (95% CI)	*p-value*
**Mullen Scale of Early learning test**					
Low visual reception score	10 (8.3)	4 (17.4)	6 (6.2)	3.2 (0.8, 14.0)	0.10
Low ELC* score	23 (19.2)	5 (21.7)	18 (18.6)	1.2 (0.4, 3.8)	0.77
**Composite outcome**					
Abnormal Visual Function**	9 (7.5)	4 (17.4)	5 (5.2)	3.8 (0.9, 15.2)	0.07
Visually Impairment	17 (14.2)	7 (30.4)	10 (10.3)	3.7 (1.2, 11.0)	0.02

*ELC stands for Early learning composite

**See details in S5 Table

## Discussion

We evaluated the outcome of visual function in young children exposed to ZIKV *in utero* in Nicaragua by ophthalmic examination and the MSEL Visual Reception scale. Comprehensive ophthalmic examination showed no structural abnormalities consistent with ocular manifestations of ZIKV infection in our cohort, and the differences between the groups in the MSEL subscale of visual reception, while lower in ZIKV-exposed children, were not statistically significant. Visual impairment (a composite score defined as either a low visual reception MSEL score or any adverse finding on the functional eye exam), was more common in ZIKV-exposed children, but, again the differences were not statistical significant.

Our study did not find a difference in visual acuity between ZIKV-exposed and ZIKV-unexposed infants without microcephaly or ocular structural lesions by preferential looking using the Lea grating test. Multiple studies have found differences in visual acuity between infants with CZS and age-matched controls [11,17,21,26,27], even those without microcephaly or ocular lesions. There are fewer data regarding visual acuity and visual function in ZIKV-exposed children that do not meet criteria for CZS and do not have retinal lesions. In Sao Paolo, Brazil, Portnoi Baran and colleagues found that the visual acuities of most children in their subgroup of ZIKV-exposed infants without microcephaly were within the normative tolerance limits [17]. However, a correlation analysis suggested that visual acuity development may have been delayed compared to unexposed controls. Similarly, Lima et al., [28] found that visual acuity for ZIKV-exposed children without clinical symptoms did not differ from normal visual acuity values for age. Their study performed multiple repeated measurements of acuity over time to evaluate visual development trajectories and separate visual acuity measurement variability as a cofounding factor. It is possible that our result of no visual acuity differences between ZIKV-exposed and unexposed groups reflects findings similar to those studies; however, we only tested visual acuity at one-time point and cannot determine the reproducibility of our measurement nor make conclusions about developmental trajectories.

Most infants that met criteria for abnormal visual function did so by displaying decreased contrast sensitivity on the Hiding Heidi test. There were no statistically significant differences between ZIKV-exposed and unexposed groups with respect to low ELC scores (*p* = 0.77) or low visual reception scores (*p* = 0.10), though ZIKV-exposed infants were more likely to perform poorly on the visual reception domain of the MSEL. When the MSEL visual reception score was combined with the visual function measure for a composite outcome of visual impairment, the difference between the groups reached statistical significance (*p* = 0.02). This suggests that a combination of the neurological and ophthalmological evaluations would be a better approach to assess subtle changes in children visual health caused by ZIKV exposure during the fetal life. Sensitivity analysis under the assumption that either all children with unknown ZIKV status were ZIKV-exposed *in utero* or under the assumption that none of them were exposed did not change the observed association (S3 and S4 Tables)

As previously mentioned, the infants that met criteria for abnormal visual function did so predominantly by showing decreased contrast sensitivity. Decreased contrast sensitivity amongst ZIKV-exposed infants is well-described [8,11,20]. Contrast sensitivity does not have a strong value as a sole diagnostic or screening test in ophthalmology, but it is recommended as part of a functional visual assessment when there are concerns for low vision or assessment of disability. In adults, contrast sensitivity is thought to be related to reading fluency, mobility performance, and performing activities of daily living [29]. Inability to recognize lower levels of contrast on the test may correlate with the infant having difficulty recognizing details in facial features, an important visual developmental milestone (age 7–10 months) and tenant of infant communication.

The MSEL visual reception test is a neurodevelopmental assessment for normative visual milestones as well as visual spatial analysis and problem solving according to a child’s developmental age [30]. In early childhood, neurodevelopmental psychologists have identified two main components of working memory: auditory-verbal and visuo-spatial [31]. Visual-spatial memory is essential for children to understand, learn from, and participate in their physical environment [32]. The combination of contrast assessment and the MSEL’s assessment of visual reception may provide a more comprehensive evaluation of visual impairment in ZIKV-exposed infants. This may be of particular importance in ZIKV-exposed children with a “mild phenotype” who perform well overall on comprehensive neurodevelopmental assessments but can show subtle visual-spatial deficiencies which may affect development and later school performance.

Congenital CMV and *Toxoplasma* infections have been associated with abnormal visual function [33,34]. Thus, to rule out any potential bias or misclassification, CMV- and *Toxoplasma*-IgM were tested in umbilical cord serum. All newborns were CMV IgM-negative and only 1.7% were *Toxoplasma* IgM-positive; therefore, CMV and/or Toxoplasmosis infection were unlikely to have significantly impacted our observation that children exposed to ZIKV *in utero* are more likely to have impaired visual function.

Our study was limited by a sample size of 159 children, of which 33 (20.8%) could not be classified with respect to ZIKV-exposure, so we were underpowered to detect subtle differences between groups. Another limitation of this study is the lack of longitudinal follow-up of visual functioning, as a cross-sectional evaluation does not allow us to draw conclusions about developmental trajectories. There is previous evidence suggesting that visual acuity losses only occur in infants who suffered gestational infection, not simply exposure [17]. Thus, a limitation of the current study is that we were unable to systematically test for ZIKV infection all the children in this study. The study groups being compared in this manuscript derive from our previously published work on maternal ZIKV serostatus, which was robust in identifying women pregnant at the time of peak ZIKV transmission in Nicaragua [21]. For children, ZIKV exposure is a pre-requisite for ZIKV infection. Our classification provided an effective heuristic for assigning likelihood of maternal ZIKV infection during pregnancy.

A strength of our study is the data on ocular outcomes in children exposed to ZIKV *in utero* without evidence of CZS at birth. Most studies in Latin American have focused on children born with overt clinical signs of congenital ZIKV infection, though some studies have included normo-cephalic children in their analyses [16,34–36]. Most children in our study demonstrated normal visual function; however, although limited by a small sample size, we do show that compared to unexposed children, children exposed to ZIKV *in utero* are more likely to have impaired visual function, particularly contrast sensitivity. These impairments could have implications for later academic performance, specifically reading. Our results provide evidence that all children exposed to ZIKV during gestation should be followed long-term during childhood, with particular attention to their visual performance and neurodevelopment. Future studies may consider the use of ZIKV serology in cord blood samples as evidence of ZIKV exposure during pregnancy to assess the risk of visual impairment in children after ZIKV epidemics.

This study supports previous recommendations [22] that all ZIKV-exposed children, even those without obvious physical or neurodevelopmental abnormalities, should undergo comprehensive evaluation including a complete eye exam, as they may demonstrate subtle yet developmentally important visual impairment that may translate to abnormal visual milestones and later challenges with academic performance.

## Supporting information

S1 Text Childbirth summary. (DOCX)

S1 Table Definition of serological status in utero. (DOCX)

S2 Table Mullen’s Visual Reception task list according to the age range and the score obtained in aech task. (DOCX)

S3 Table Ocular finding of children exposed, unexposed and unknown status of ZIKV in a cohort in León, Nicaragua, 2016–2017. (DOCX)

S4 Table All unknown status children have ZIKV exposure / No unknown status children have ZIKV exposure. (DOCX)

S5 Table Description of the variables that were taken into account to define an Abnormal Visual Function and Visual Impairment. (DOCX)
