# Supplementary material for: Visual findings in children exposed to Zika in utero in Nicaragua
Source: PLoS Negl Trop Dis. 2023 May 19;17(5):e0011275. doi: 10.1371/journal.pntd.0011275 (PMC10234517; doi:10.1371/journal.pntd.0011275)
Supplement: S1 Text — (DOCX) [file pntd.0011275.s001.docx]

**S1 Text. Childbirth summary**

**Resumen del Parto**

1. Número de identificación del paciente (Maternal study ID) : ______________________

2. Fecha del parto (Date of delivery):________________________________ Día/Mes/Año (DD/MM/YY)

3. Edad gestacional en el momento del parto (Infant gestational age at time of delivery): ________Semanas (Weeks) ________Días (Day)

4. Peso del bebe (Infant birthweight): ____________gramos (grams)

5. Sexo (Sex of infants): _________Masculino (male) _________Femenino (female)

6. Apgar 1 min: _________/10_______ 5 min: _________/10_______

7. Modo de parto (Mode of delivery): _________Parto normal vaginal (SVD) _________Cesárea (Caesarian)

7a. Si fue cesárea (If caesarean): _________electiva programada (elective repeat) _________emergencia (Unscheduled)

7b. Si fue una cesárea inesperada (If unscheduled):

Dilatación cervical en el momento de cesárea (cervical dilation at time of CS): ____________cm

8. Duración del parto (Length of time in labor): ___________horas (Hours) __________ cesáreo electivo (NA patient had elective CS)

9. ¿La membranas se rompieron antes del parto(Membranes ruptured before delivery)? Si (Yes)_________ No (No)________

9a: En caso afirmativo, ¿cuánto tiempo antes del parto se rompieron? (If yes, how long were membranes ruptured before delivery) _________horas (Hours)

10. ¿Hubieron infecciones en el parto? (Were there any infections diagnosed in labor?) Si (Yes)________ No (No)________

11. ¿Se trataron con antibióticos? (If yes, was the infection treated with antibiotics?) Si (Yes)________ No (No)________

1. ¿Qué tipo de infección fue y que antibióticos fueron suministrados? (What type of infection was it and what antibiotics were given?)

___________________________________________________________________________________________________________________________________________________________________________________________________________________________________________________________________________________________________

12. Disposición del bebé (Where did the infant go after delivery?):

_______Con la madre (With mother) _______unidad de cuidados intensivos (Intensity care unit) ________muerte fetal (stillbirth)

1. En caso de cuidados intensivos o muerte fetal, ¿cuál fue la razón? (¿In case of intensive care or stillbirth, what was the reason?)

_________________________________________________________________________________________________________________________________________________________________________________

1. Edad del bebe al momento de muerte fetal (Age of the baby at the time of fetal death): _____________ Días (Days)

13. Examen físico infantil (Child Physical Exam):

1. Circunferencia de la cabeza (cm) (Head circumference of infant): _______________
2. Talla (cm) (Length of infant): __________
3. Esplenomegalia (splenomegaly): SI (Yes)_______ NO (No) ________
4. Hepatomegalia (hepatomegaly): SI (Yes)_______ NO (No)________
5. Erupción Cutánea (skin rash)): SI (Yes)_______ NO (No)________

14. ¿Alguna anomalía del bebé?( Any abnormalities of infant?) Si_________ No____________

14a. Si la respuesta es afirmativa, por favor describa (If yes please describe):

__________________________________________________________________________________________________________________________________________________________________________________________________________________________________________________________________________________________________

15. Examen neurológico del bebé antes de alta hospitalaria (Neurological examination of the baby before hospital discharge):

1. Hipotonía (Hypotony): SI (Yes) _______ NO (No) ________
2. Hipertonía/Espasticidad (Hypertonia): SI (Yes) _______ NO (No) ________
3. Ojos capaces de rastrear (eyes capable of tracking): SI (Yes) _______ NO (No) ________
4. Hiperreflexia (hyperreflexia): SI (Yes) _______ NO (No) ________
5. Irritabilidad (irritability): SI (Yes) _______ NO (No) ________
6. Convulsiones/Tremores (seizures): SI (Yes) _______ NO (No) ________
7. Microcefalia (microcephaly): SI (Yes) _______ NO (No) ________
8. Alguna otra normalidad neurológica (Describa) (Any other neurological abnormality, describe):

___________________________________________________________________________________________________________________________________________________________________________________________________________________________________________________________________________

16. ¿Algún hallazgo anormal en el ultrasonido durante el embarazo? (Were there any abnormal US findings during pregnancy, describe)

Si (Yes)____________ No (N**o**)___________

16a. Si la respuesta es afirmativa, por favor describa (If yes, please describe):

__________________________________________________________________________________________________________________________________________________________________________________________________________________________________________________________________________________________________
