# Supplementary material for: Visual findings in children exposed to Zika in utero in Nicaragua
Source: PLoS Negl Trop Dis. 2023 May 19;17(5):e0011275. doi: 10.1371/journal.pntd.0011275 (PMC10234517; doi:10.1371/journal.pntd.0011275)
Supplement: S1 Table — (DOCX) [file pntd.0011275.s002.docx]

**S1 Table Definition of ZIKV serological status in utero.**

| **Status** | **Definition** |
| --- | --- |
| **ZIKV Exposed** | Defined as meeting any of the following:  1. High maternal serum titers (FRNT^a^>3000) to ZIKV at the time of birth.  2. A 4-fold or greater increase in maternal serum FRNT50 value between prenatal and delivery samples.  3. Detection of anti-ZIKV IgM in any maternal or umbilical cord blood serum sample. |
| **ZIKV Unexposed** | Infants were considered unexposed if maternal serology was consistent with being ZIKV-naïve or immune to ZIKV before conception, defined as:  1. ZIKV levels of FRNT50 between 40 and 300 and stable throughout pregnancy.  2. No detectable ZIKV-specific IgG  3. FRNT or eFRNT50 less than 200. |

^a^ FRNT: focus reduction neutralization test
