# Supplementary material for: Visual findings in children exposed to Zika in utero in Nicaragua
Source: PLoS Negl Trop Dis. 2023 May 19;17(5):e0011275. doi: 10.1371/journal.pntd.0011275 (PMC10234517; doi:10.1371/journal.pntd.0011275)
Supplement: S2 Table — (DOCX) [file pntd.0011275.s003.docx]

**S2 Table: Mullen's Visual Reception task list according to the age range and the score obtained in each task**

| **Age in months** | **Task** | **Score** |
| --- | --- | --- |
| **13-20** | 9. Look for the ring under the cloth  (1) Partially hidden (2) Completely hidden | 2; 1; 0 |
|  | 10. Straighten the cup | 1;0 |
|  | 11. Makes object association  ___ brush ___ spoon ___ cup ___ ball | 1;0 |
|  | 12. Look for the cart under two cloths | 1;0 |
|  | 13. Shows interest in the book as a hinge | 1;0 |
|  | 14. Pay attention to the drawing | 1;0 |
| **23-32** | 15. Look for the covered toy, then displaced | 1;0 |
|  | 16. Can discriminate between shapes on the board  (1) circle (2) circle and square (3) circle, square, triangle (4) circle, square, triangle, + | 4; 3; 2; 1; 0 |
|  | 17. A. Matches objects to their name (19 months or younger)  EITHER  17B. Match objects without naming them (20 months or older)  __ shoe ___ cars ___ keys ___ sticks  (1) An object with your name on it  (2) 2 objects without naming them  (3) 3 objects without naming them | 3; 2; 1; 0 |
|  | 18. Putting one cup inside another | 2; 1; 0 |
