# Supplementary material for: Visual findings in children exposed to Zika in utero in Nicaragua
Source: PLoS Negl Trop Dis. 2023 May 19;17(5):e0011275. doi: 10.1371/journal.pntd.0011275 (PMC10234517; doi:10.1371/journal.pntd.0011275)
Supplement: S3 Table — (DOCX) [file pntd.0011275.s004.docx]

**S3 Table. Ocular finding of children exposed, unexposed and unknown status of ZIKV in a cohort in León, Nicaragua, 2016-2017.**

|  | **All Participants (%)**  **N=157** | **ZIKV Exposed (%)**  **N=24** | **ZIKV Unexposed (%)**  **N=100** | **Unknown Exposure Status (%) N=33** | **Odds Ratio**  **(95% CI) ^a b^** | **Mean Difference**  **(95% CI) ^a^** | **p-value** |
| --- | --- | --- | --- | --- | --- | --- | --- |
| **Functional eye exam** | | | | | | | |
| No change of gaze between 2 objects at 30 cm | 4 (2.5) | 1 (4.2) | 3 (3.0) | 0 (0) | 1.3 (0.0 – 12.5) | ------- | >0.99 |
| Hiding Heidi Contrast greater than 5% | 6 (3.8) | 3 (12.5) | 2 (2.0) | 1 (3.0) | 6.9 (1.0 – 57.0) | ------- | 0.05 |
| **Visual acuity** | | | | | | | |
| Abnormal visual field | 0 (0) | 0 (0) | 0 (0) | 0 (0) | ------- | ------- | ------- |
| Abnormal accommodative reflex | 0 (0) | 0 (0) | 0 (0) | 0 (0) | ------- | ------- | ------- |
| Lea visual acuity, cpcm, mean (SD) |  |  |  |  |  |  |  |
| OD | 4.6 (1.9) | 4.5 (1.9) | 4.6 (1.9) | 4.7 (1.9) | ------- | -0.1 (-1.2 – 1.0) | 0.86 |
| OS | 4.7 (2.0) | 4.5 (1.9) | 4.7 (2.0) | 4.8 (1.9) | ------- | -0.2 (-1.3 – 0.9) | 0.71 |
| OU | 6.2 (2.8) | 6.3 (3.3) | 6.0 (2.8) | 6.6 (2.3) | ------- | 0.3 (-1.0 – 1.6) | 0.65 |
| No fix and follow | 2 (1.3) | 0 (0) | 2 (2.0) | 0 (0) | 0.0 (0.0 – 15.1) | ------- | >0.99 |
| No reaction to light | 2 (1.3) | 0 (0) | 2 (2.0) | 0 (0) | 0.0 (0.0 – 14.5) | ------- | >0.99 |
| **External eye** | | | | | | | |
| Abnormal motility | 0 (0) | 0 (0) | 0 (0) | 0 (0) | ------- | ------- | ------- |
| Strabismus | 1 (0.6) | 0 (0) | 1 (1.0) | 0 (0) | 0.0 (0.0 – 79.2) | ------- | >0.99 |
| Abnormal external eye | 7 (4.5) | 0 (0) | 6 (6.0) | 1 (3.0) | 0.0 (0.0 – 3.1) | ------- | 0.60 |
| Abnormal anterior segment | 2 (1.3) | 0 (0) | 2 (2.0) | 0 (0) | 0.0 (0.0 – 16.8) | ------- | >0.99 |
| **Evaluation summary** | | | | | | | |
| Abnormal functional sight | 2 (1.3) | 1 (4.2) | 1 (1.0) | 0 (0) | 4.3 (0.1 – 167.3) | ------- | 0.35 |
| Abnormal refraction | 1 (0.6) | 0 (0) | 1 (1.0) | 0 (0) | 0.0 (0.0 – 78.4) | ------- | >0.99 |
