# Supplementary material for: Visual findings in children exposed to Zika in utero in Nicaragua
Source: PLoS Negl Trop Dis. 2023 May 19;17(5):e0011275. doi: 10.1371/journal.pntd.0011275 (PMC10234517; doi:10.1371/journal.pntd.0011275)
Supplement: S4 Table — (DOCX) [file pntd.0011275.s005.docx]

**S4 Table. All unknown status children have ZIKV exposure**

**Composite outcome of visual impairment**

|  | **All participants (%) N=157** | **ZIKV Exposed (%) N=57** | **ZIKV Unexposed (%) N=100** | **Odds Ratio (95% CI) ^a^** | ***p-value*** |
| --- | --- | --- | --- | --- | --- |
| **Visually Impaired** ^b^ | 23 (14.6) | 13 (22.8) | 10 (10.0) | 2.7 (1.1, 6.5) | 0.03 |
| **Abnormal Visual Function** ^c d^ | 10 (6.4) | 5 (8.8) | 5 (5.0) | 1.8 (0.5, 6.6) | 0.50 |

^a^ Comparisons are between those with incident infections and those with no incident infection

^b^ Includes any adverse findings on the functional eye exam or low visual reception score

^c^ Odds ratio confidence intervals and p values calculated using Fisher’s Exact Test and minimum likelihood principle

^d^ Includes any adverse findings on the functional eye exam

**No unknown status children have ZIKV exposure**

**Composite outcome of visual impairment**

|  | **All participants (%) N=157** | **ZIKV Exposed (%) N=24** | **ZIKV Unexposed (%) N=133** | **Odds Ratio (95% CI) ^a b^** | ***p-value*** |
| --- | --- | --- | --- | --- | --- |
| **Visually Impaired** ^c^ | 23 (14.6) | 7 (29.2) | 10 (7.5) | 3.0 (1.0, 8.4) | 0.05 |
| **Abnormal Visual Function** ^d^ | 10 (6.4) | 4 (16.7) | 6 (4.5) | 4.2 (1.0, 18.3) | 0.05 |

^a^ Comparisons are between those with incident infections and those with no incident infection

^b^ Odds ratio confidence intervals and p values calculated using Fisher’s Exact Test and minimum likelihood principle

^c^ Includes any adverse findings on the functional eye exam or low visual reception score

^d^ Includes any adverse findings on the functional eye exam
