# Supplementary material for: Visual findings in children exposed to Zika in utero in Nicaragua
Source: PLoS Negl Trop Dis. 2023 May 19;17(5):e0011275. doi: 10.1371/journal.pntd.0011275 (PMC10234517; doi:10.1371/journal.pntd.0011275)
Supplement: S5 Table — (DOCX) [file pntd.0011275.s006.docx]

**S5 Table. Description of the variables that were taken into account to define an Abnormal Visual Function and Visual Impairment.**

| **Parameters** | **# (%) of children ZIKV exposed, n = 23** | **# (%) of children Unexposed, n = 97** |
| --- | --- | --- |
| - **Abnormal visual function** |  |  |
| - - Shift of gaze testing negative | 1 (4.4) | 3 (3.1) |
| - - Abnormal Confrontational visual field testing | 0 (0) | 0 (0) |
| - - Abnormal accommodative reflex | 0 (0) | 0 (0) |
| - - 5% or lower Hiding Heidi test did not elicit a response | 3 (13.0) | 2 (2.1) |
| - - All children | 4 (17.4) | 5 (6) |
| - **Low ELC score in the subdomain of visual reception of MSEL*** | 4 (17.4) | 6 (6.2) |
| **Visual impairment Composite** | 7 (30.4) | 10 (10.3) |

ELC and MSEL stands for Early Learning Composite and Mullen scale of Early Learning, respectively.

*A visual reception subdomain score of ≤ 39
